# Impacts of morphine addiction on spermatogenesis in rats

**Published:** 2016-05

**Authors:** Nasrin Takzare, Esmaeil Samizadeh, Saeed Shoar, Masoumeh Majidi Zolbin, Mohammad Naderan, Ali Lashkari, Azam Bakhtiarian

**Affiliations:** 1 *Department of Anatomy, Tehran University of Medical Sciences, Tehran, Iran.*; 2 *Department of Pathology, Tehran University of Medical Sciences, Tehran, Iran.*; 3 *Department of Surgery, Shariati Hospital, Tehran University of Medical Sciences, Tehran, Iran.*; 4 *Department of Pharmacology, Faculty of Medicine, Tehran University of Medical Sciences, Tehran, Iran.*

**Keywords:** *Opioids*, *Morphine*, *Spermatogenesis*, *Sperm*, *Rats*

## Abstract

**Background:**

There are numerous investigations on wide range of issues that disrupt regulatory spermatogenesis, individuals who are exposed to drug abuse faced infertility and immature spermatogenesis.

**Objective:**

The aim of this study was to evaluate the addiction effects of morphine and its derivatives on rats spermatogenesis.

**Materials and Methods:**

40 male Wistar rats were randomly divided into 5 equal groups, which were exposed either with intravenous morphine, naloxone, naloxone and morphine, sham (with normal saline injection) and a control group without infusion. Spermatogenesis was assessed after three months via histological sections with hematoxylin and eosin staining, using a light microscope based on measurement of spermatogonia, spermatocyte, spermatid, and spermatozoa.

**Results:**

Those rats that received opioids had changes in spermatogenesis function. The population of spermatogenesis cycle cells at spermatogonia, spermatocyte, spermatid, and spermatozoa stages was significantly decreased in those rats that received opioid in comparison to the control group (p<0.05). Histological studies revealed that changes in different groups of opioid application might affect sperm formation. Sperm count in morphine group was (0±0) and in naloxone group, naloxone+morphine, sham and control were 235±3.77, 220±3.81, 247.12±6.10 and 250±6.54, respectively (p<0.001).

**Conclusion:**

Morphine could affect all spermatogenesis stages.

## Introduction

Despite the large number of studies addressing the impacts of opioid consumption in women on pregnancy outcomes, few have found such an effect on male fertility along with paternal exposure relationship in development of offspring physiological and behavioral features (1-3). In some cases opioid drugs are used as pain reliever or to treat premature ejaculation, which may affect fertility (4). Herbal drugs such as Achillea millefolium or environmental factors such as Bisphenol-A might have same function and affect germinal epithelium in gonadal region (5). However, due to many involved cofounding variables, primary explanation for this relationship has never been well studied (1).

In contrast to the human subjects, animal models are available, allowing for more robust and much more biological assessment of underlying processes (1, 6-9). With this in mind, extensive amounts of evidence have supported the relationship between chronic exposures of adult male rodents to opiates with development of future generation (6, 10-13). No single study has been done to evaluate the exact mechanism of this phenomenon yet. Many studies have shown the effect of opioid on hypothalamic and gonadal system via evaluating gene expression and hormonal analysis (14, 15). However, the results are not conclusive because of lacking morphological studies. 

Therefore, we aimed to evaluate the histological alterations in different stages of spermatogenesis in male rats. For this purpose, we evaluated adult male rat testis under a chronic exposure to opioids such as morphine to reveal the fertility status on paternity of animal model.

## Materials and methods

**Animal model establishment**

This experimental animal study was conducted on forty adult male Wistar rats obtained from Pasteur Institute Iran with a mean age of three months and a weight range of 250-300 gr. The ethics committee of Tehran University of Medical Sciences approved the study and all protocols provided by Institutional Committee of Animal Care were exactly followed. 

The rats were housed singly in a room in animal laboratory at 12 hr light/dark cycle provided with proper nutrition (Pars Animal Feed Factory, Iran). Temperature was set between 20-24^o^C. The rats were randomized into five equal groups (8 rats in each group) as follows: morphine, naloxone, naloxone and morphine, sham, and control. Sham group was injected only with normal saline serum and control group was only observed without any type of injection. 

Morphine sulfate (TEMAD, Iran) was injected subcutaneously (5 mg/kg) and its effects was evaluated in 7 different time intervals, i.e. days 0, 10, 20, 30, 40, 70, 90 (16, 17). Because of short and long term effects of morphine, we evaluated it’s early and late effects by choosing these different timelines (18, 19). Withdrawal symptoms were checked subsequently including tremor, movements of limb off the baseline floor or diarrhea occurrence after 30 min of injection. Naloxone (Darupakhsh, Iran) which was chosen as antagonist for morphine addiction was administrated 1.5 mg/kg intra-peritoneally (20). Naloxone was injected 30 min before the test (17). Moderate dose of naloxone and morphine were administered in the naloxone+morphine group (1, 4 respectively), and normal saline was administered in the sham group (17).

**Histological study**

According to the mentioned timeline, each group of rats was scarified by chloroform and testes were removed and washed out for histological analysis. Tissue samples were added to fixative solutions (formalin) for 24 hr. The tissues were washed with alcohol, embedded in paraffin, sliced in a 5 µm thickness and finally stained with hematoxylin and eosin (Sigma, England). Using a light microscope (CX31- OLYMPUS, Japan) and a magnification field of 40×/0.65 to evaluate spermatogenesis in different stages. 

**Morphometry of spermatogenesis cycle in testis**

Through the seminiferous location characterization, we can evaluate spermatogenesis stages in seminiferous tubules. In each cross section of seminiferous tubule, stage analysis was done through the tubules random selection. Cells were counted according to the shape of nuclei and their location to luminal part and were evaluated by Photo tools version 2 (Microsoft Corp.) (21). 

**Statistical analysis**

Data were analyzed by IBM statistical package for the social sciences (SPSS, version 21, IBM Corp., New York, US) to show the fertility indices difference between experimental groups. Normal distribution of the data was evaluated using Kolmogorov-Smirnov test. In order to analyze continuous variables, t-test and one-way ANOVA were applied for parametric variables while Kruskal-Wallis and Mann-Whitney U-tests were used to analyze the nonparametric variables. The obtained values were deemed significant at p<0.05.

## Results

**Figure 1:**
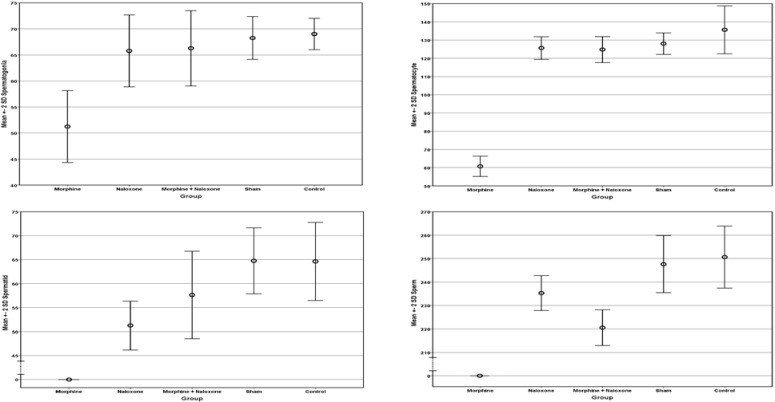
Comparison of the means of the spermatogenesis cycle cells counts according to different study groups

**Figure 2:**
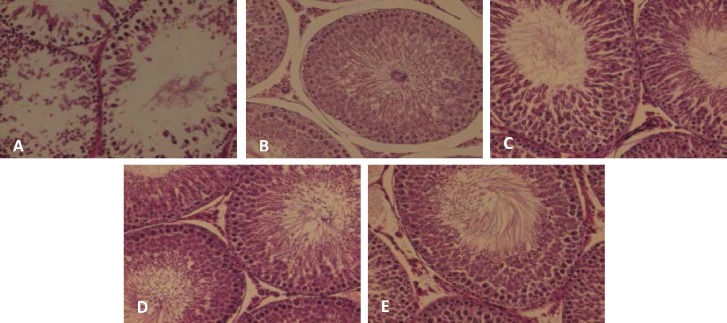
Microscopic view of seminiferous tubules.

There were no casualties in rats. Fertility parameters characteristics and measurements are summarized in table I. Figure 1 also demonstrates parameters mean±SD according to different groups. The histological evaluation of spermatogenesis cycle in each group is shown in figure 2. The intergroup comparisons of different study groups according to fertility parameters are shown in table II. As clearly indicated, the morphine group had a statistically significant difference with all other groups in term of spermatogonia, spermatocyte, spermatid, and sperm counts, and also testes weight.

**Table I:** Comparison of the different spermatogenesis cells counts between study groups (8 rats in each group

**Parameters **	**Morphine**	**Naloxone**	**Morphine + Naloxone**	**Sham**	**Control**	**p-value**
Spermatogonia	51 ± 3.46	65 ± 3.46	66 ± 3.62	68 ± 2	69 ± 1.40	<0.001
Spermatocyte	60 ± 2.77	125 ± 3.16	124 ± 3.58	128 ± 2.87	135 ± 6.54	<0.001
Spermatid	0±0	51 ± 21.59	57 ± 4.59	64 ± 3.46	64 ± 4.06	<0.001
Sperm	0±0	235 ± 3.77	220 ± 3.81	247 ± 6.10	250 ± 6.54	<0.001
Testes Weight (mg)	1.12 ± 0.04	1.35 ± 0.03	1.32 ± 0.01	1.38 ± 0.02	1.39 ± 0.05	<0.001

Data are presented as mean±SD. Mann-Whitney U test was used.

**Table II:** Intergroup Comparisons of different study groups according to fertility parameters

**Parameters **	**M-N**	**M-M+N**	**M-S**	**M-C**	**N-M+N**	**N-S**	**N-C**	**M+N-S**	**M+N-C**	**S-C**
Spermatogonia	0.001	0.001	0.001	0.001	0.526	0.064	0.011	0.187	0.030	0.151
Spermatocyte	0.001	<0.001	0.001	0.001	0.526	0.081	0.004	0.039	0.002	0.030
Spermatid	<0.001	<0.001	<0.001	<0.001	0.532	0.013	0.011	0.004	0.003	0.741
Sperm	<0.001	<0.001	<0.001	<0.001	<0.001	<0.001	<0.001	<0.001	<0.001	0.372
Testes Weight	0.001	0.001	<0.001	<0.001	0.442	0.092	0.026	0.003	<0.001	0.652

Data are presented as mean±SD. Mann-Whitney U test was used.

M: Morphine

N: Naloxone

M+N: Morphine + Naloxone

S: Sham

C: Control.

A)Morphine group. B) Control group. C) Sham group. D) Naloxone group. E) Naloxone+Morphine group (Staining, H&E ×40).

## Discussion

Opioid can be applied for medical purposes; also in current decade, its prevalence usage among young people in society led us to run out this work to represent its effect on male reproductive system via spermatogenesis evaluation. The opioid system as a biological exchange apparatus is modulated by opioid peptides (22). These endogenous peptides play an important role in the control of pituitary luteinizing hormone (LH) secretion, acting on releasing of gonadotropin hormone (GnRH) release from the hypothalamus (23, 24). Administration of exogenous opiates such as morphine has been shown to decrease the levels of LH in contrast to blockade of endogenous opioid peptide receptor (25).

Wang *et al* studied the effect of heroin addiction on pituitary-testicular function. They indicated that testicular function had decreased in opioid addiction (26). Some other studies evaluated the effect of opioids on other organs, the most important on cerebellar structure, and found these compounds increase the neurotoxic factors and seems to disrupt the main neural tract (27). In the literature, multiple cases of toxicity and abuse of opioid like morphine and tramadol have been reported, and they figured out these drugs effect on reproductive dysfunction, our findings are in line with recent studies carried out by El Sawy *et al* and El-Ghawet *et al* who reported that injections of tramadol would face with disorganization of seminiferous tubules with almost missing of fertility and comparatively decreased spermatogenic cells in rats (28).

Singer *et al* reported that application of hashish, heroin or morphine has direct effect on vitality, morphology and motility of sperm. They evaluated the semen smear and observed that 20-30% of spermatozoa had weak motility, 40% of spermatogenic cells had pathologic abnormality with decreased number. Finally, they found that morphine family drugs result in oligospermia and azoospermia (29). Furthermore, administration of tramadol and morphine lead to structural abnormalities and could disrupt the normal histological structure of rat testis (30). Our study indicated that all spermatogenic cell populations had decreased levels under the effect of chronic exposure to morphine sulphate. Same results have been already reported by James and colleagues that 13 weeks after withdrawing morphine sulfate the quantitative reductions in the population of spermatogenic cells and levels of pituitary gland hormones were reversed (31). 

In addition, in the current study, administration of naloxone was associated with modification of spermatogenic cells count compared with previously exposure to morphine sulphate. Nevertheless, the cell counts did not reach to physiologic level in each group. This could be explained by the results of Bablok *et al* in which elevation of serum levels of LH, FSH, prolactin, and estradiol were observed while the amount of free testosterone was significantly diminished after performing of naloxone test administering 0.4 mg naloxone intravenously (32). However, in contrast with present study, Cicero *et al* have concluded that sperm counts and motility are not affected by administration of morphine sulphate (1). In addition, the response of LH to naloxone seems to be related to the concentrations of circulating gonadal steroids in healthy men and women (25).

Effects of morphine and other opiates on reproductive system as well as germ cells and gonadal cell population have been thoroughly studied. Many alterations in reproductive systems of rats have been reported about opioid derivatives. The lower pregnancy rate in female rats mated with opiate exposed male seems to be affected by decreasing effects of morphine on sex organ secretions including seminal vesicles and prostate which together provide a transporting pathway for sperm cells (33-35). It seems that morphine and its families could act directly on opioid receptors and have a negative regulatory impact on serum levels of sex and other gonadal hormones at the same time. In addition, direct effect of opiates on morphology and function of sperm and its progenitors should never be neglected.

It is of interest if further studies evaluate morphology and motility of sperm cells.In addition, evaluating the effects of opioid derivatives on other part of reproductive system can declare the main reason of infertility.

## Conclusion

In conclusion, we showed that exposure to morphine reduces the number of all spermatogenesis cell population in male rats. In addition, administration of naloxone would have modulatory effect on spermatogenic cells population.

## Note

***This article extracted from M.D. thesis. (Ali Lashkari)***
